# *Agrobacterium rhizogenes*-mediated marker-free transformation and gene editing system revealed that *AeCBL3* mediates the formation of calcium oxalate crystal in kiwifruit

**DOI:** 10.1186/s43897-023-00077-w

**Published:** 2024-01-02

**Authors:** Pengwei Li, Yiling Zhang, Jing Liang, Xufan Hu, Yan He, Tonghao Miao, Zhiyin Ouyang, Zuchi Yang, Abdul Karim Amin, Chengcheng Ling, Yize Liu, Xiuhong Zhou, Xiaoran Lv, Runze Wang, Yajing Liu, Heqiang Huo, Yongsheng Liu, Wei Tang, Songhu Wang

**Affiliations:** 1 Anhui Provincial Key Laboratory of Horticultural Crop Quality Biology, School of Horticulture, Anhui Agricultural University, Hefei, 230036 China; 2 Mid-Florida Research and Education Center, University of Florida, Institute of Food and Agricultural Sciences, Apopka, FL 32703 USA

**Keywords:** Marker-free transformation, *Agrobacterium rhizogenes*, CRISPR/Cas9, Kiwifruit, *AeCBL3*

## Abstract

The transformation and gene editing of the woody species kiwifruit are difficult and time-consuming. The fast and marker-free genetic modification system for kiwifruit has not been developed yet. Here, we establish a rapid and efficient marker-free transformation and gene editing system mediated by *Agrobacterium rhizogenes* for kiwifruit. Moreover, a removing-root-tip method was developed to significantly increase the regeneration efficiency of transgenic hairy roots. Through *A. rhizogenes*-mediated CRISPR/Cas9 gene editing, the editing efficiencies of *CEN4* and *AeCBL3* achieved 55 and 50%, respectively. And several homozygous knockout lines for both genes were obtained. Our method has been successfully applied in the transformation of two different species of kiwifruit (*Actinidia chinensis* ‘Hongyang’ and *A.eriantha* ‘White’). Next, we used the method to study the formation of calcium oxalate (CaOx) crystals in kiwifruit. To date, little is known about how CaOx crystal is formed in plants. Our results indicated that *AeCBL3* overexpression enhanced CaOx crystal formation, but its knockout via CRISPR/Cas9 significantly impaired crystal formation in kiwifruit. Together, we developed a fast maker-free transformation and highly efficient CRISPR-Cas9 gene editing system for kiwifruit. Moreover, our work revealed a novel gene mediating CaOx crystal formation and provided a clue to elaborate the underlying mechanisms.

**Graphical abstract:**

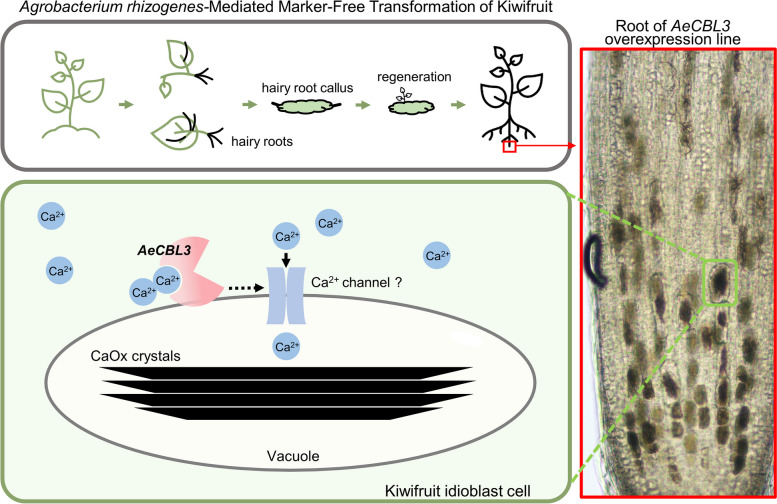

**Supplementary Information:**

The online version contains supplementary material available at 10.1186/s43897-023-00077-w.

## Core

We developed a convenient maker-free transformation and highly efficient CRISPR-Cas9 gene editing system for kiwifruit. Through the method, we demonstrated that *AeCBL3* positively mediates the formation of calcium oxalate crystals in kiwifruit.

## Gene & Accession Numbers

Genes and Sequence data used in this article can be found in the Kiwifruit Genome Database (KGD, http://kiwifruitgenome.org/) under the accession numbers: *CEN4* (DTZ79_19g06720), *Calcineurin B-like 3* gene (DTZ79_17g06240).

## Introduction

The woody species kiwifruit belongs to the genus *Actinidia* and its fruits are popular worldwide because of their high levels of vitamin C, minerals, dietary fiber, and other healthy metabolites (Sivakumaran et al. 2018). The genus includes 54 species and four species (*Actinidia chinesis*, *A. deliciosa*, *A. eriantha*, and *A. arguta*) have been developed as commercial cultivars so far. Due to its nutrient and commercial values, extensive investigations on genomics (Huang et al. 2013; Pilkington et al. 2018; Tang et al. 2019; Wu et al. 2019; Yue et al. 2023), transcriptomics (Choi et al. 2022; Li et al. 2022; Tahir et al. 2022; Miao et al. 2023; Xiong et al. 2023), and metabolomics (Abid et al. 2022; Jia et al. 2022; Wang et al. 2022a; Wang et al. 2022c; Shu et al. 2023; Zhang et al. 2023) of kiwifruit have been carried out. However, functional genomic studies (Akagi et al. 2019; Peng et al. 2019; Fu et al. 2021; Varkonyi-Gasic et al. 2021; Wang et al. 2022) are limited, partially because the genetic modification of kiwifruit is difficult and time-consuming.

To date, the *Agrobacterium tumefaciens*-mediated transformation has been established for *A. chinensis*, *A. deliciosa*, *A. eriantha,* and *A. arguta* (Uematsu et al. 1991; Janssen and Gardner 1993; Wang et al. 2006; Herath et al. 2020). However, *A. tumefaciens*-mediated transformation usually involves extremely tedious experimental procedures, months of cycles, and effective antibiotic screening, which undoubtedly poses great challenges for rapid transformation. Marker genes, which commonly encode antibiotic or herbicide resistance, are indispensable for screening rare plant cells or tissues taking foreign DNA from the untransformed ones. Marker-free transgenic crop plants are desirable because the use of maker genes commonly causes public concerns from the food safety and ecological perspective (de Vetten et al. 2003). The marker-free transformation can be realized by using co-transformation, transposable elements, chemical-induced recombination, and intrachromosomal recombination (Dale and Ow 1991; Komari et al. 1996; Ebinuma et al. 1997; Zubko et al. 2000; Zuo et al. 2001; de Vetten et al. 2003). To date, the marker-free transformation system for kiwifruit has not been developed yet.

CRISPR/Cas9-based gene editing has been applied to many crops including kiwifruit (Bortesi and Fischer 2015; Xie et al. 2015; Wang et al. 2018). The biallelic mutation of *AcCEN* or *AcCEN4* confers rapid flowering and compact developmental traits to kiwifruit (Varkonyi-Gasic et al. 2019), and the mutation of the *SyGl* gene promotes the female development of male kiwifruit (Varkonyi-Gasic et al. 2021). The success of gene editing requires effective guide RNA (gRNA). To the best of our knowledge, the rapid screening system of effective gRNAs for kiwifruit has not been reported yet.

*Agrobacterium rhizogenes*-mediated transformation (ArMT) is another option for plant genetic modification and has been applied to many species (Mehrotra et al. 2018; Gomes et al. 2019; Meng et al. 2019; Xu et al. 2020). For instance, ArMT is widely used in soybean to study the function of genes required in the biological processes of the root (Kereszt et al. 2007). Combining *A.rhizogenes-*mediated hairy root transformation with gene delivery and gene editing has become another powerful tool extensively used in molecular genetic investigation and breeding (Butler et al. 2020; Liu et al. 2022). The combination of *A. rhizogenes* hairy root and CRISPR/Cas provides an extraordinary platform for rapid, precise, easy, and cost-effective “in root” functional analysis of genes of interest in legume plants (Niazian et al. 2022). The hairy root transformation system with gene editing in cucurbit crops has successfully revealed the salt tolerance mechanism of roots (Geng et al. 2022). Recently, the cut-dip-budding delivery system using *A. rhizogenes* achieves effective transformation and gene editing without tissue culture and sterile conditions in the plant species with root-suckering ability (Cao et al. 2023). Previous studies have shown that *A. rhizogenes* may also apply to kiwifruit (Yazawa et al. 1995; Yamakawa and Chen 1996), but few studies utilizing hairy root transformation in kiwifruit have been reported, possibly because of its low transformation and regeneration efficiency.

Oxalic acid is widely distributed in plant foods, but oxalic acid has negative effects on human health (Franceschi and Nakata 2005). Oxalic acid in food can form crystal precipitation with calcium ions and other trace elements in food, hindering the body’s absorption of these minerals and increasing the risk of kidney stones (Massey et al. 1993). Oxalic acid also precipitates with calcium to form crystals in a variety of plant species (Franceschi and Nakata 2005). For plants, calcium oxalate (CaOx) crystals formation has many functions including regulation of cellular calcium concentration, detoxification of heavy metals, and protection from herbivory (Nakata 2003). Kiwifruit also accumulates CaOx crystals (Perera et al. 1990; Nguyen and Savage 2013). A previous study has shown that CaOx crystals are responsible for mouth irritation or catch when eating kiwifruit (Perera et al. 1990). The CaOx crystal-accumulating cell is called the crystal idioblast. However, little is known about the genes mediating the formation of CaOx crystal and idioblast.

In this study, we revealed the ability of *A. rhizogenes* K599 to induce transgenic hairy roots of kiwifruit without selective pressure (antibiotics). The transgenic hairy roots can be effectively induced to generate callus and shoots by using a removing-root-tip method. The application of marker-free hairy root transformation significantly shortened the transformation cycle of kiwifruit, and the transgenic hairy roots and plantlets showed efficient and stable expression of *eGFP* and *GUS*. The method has been successfully applied in the transformation of *A. eriantha* ‘White’ and *A. chinensis* ‘Hongyang’. Moreover, the combined application of this method and the gene editing system Polycistronic tRNA-gRNA(PTG)/Cas9 successfully edited the targeting genes *CEN4* of ‘Hongyang’ and *calcineurin B–like gene 3* of ‘White’ (*AeCBL3*) with a high efficiency of producing the homozygous knockout lines. Besides, our results indicated that *AeCBL3* plays an important role in mediating calcium oxalate crystal formation in the root tip. This study offers a marker-free transformation for kiwifruit and achieves rapid identification of gene functions and genetic improvement of kiwifruit.

## Results

### Hairy root induction and shoot regeneration

**Fig. 1:**
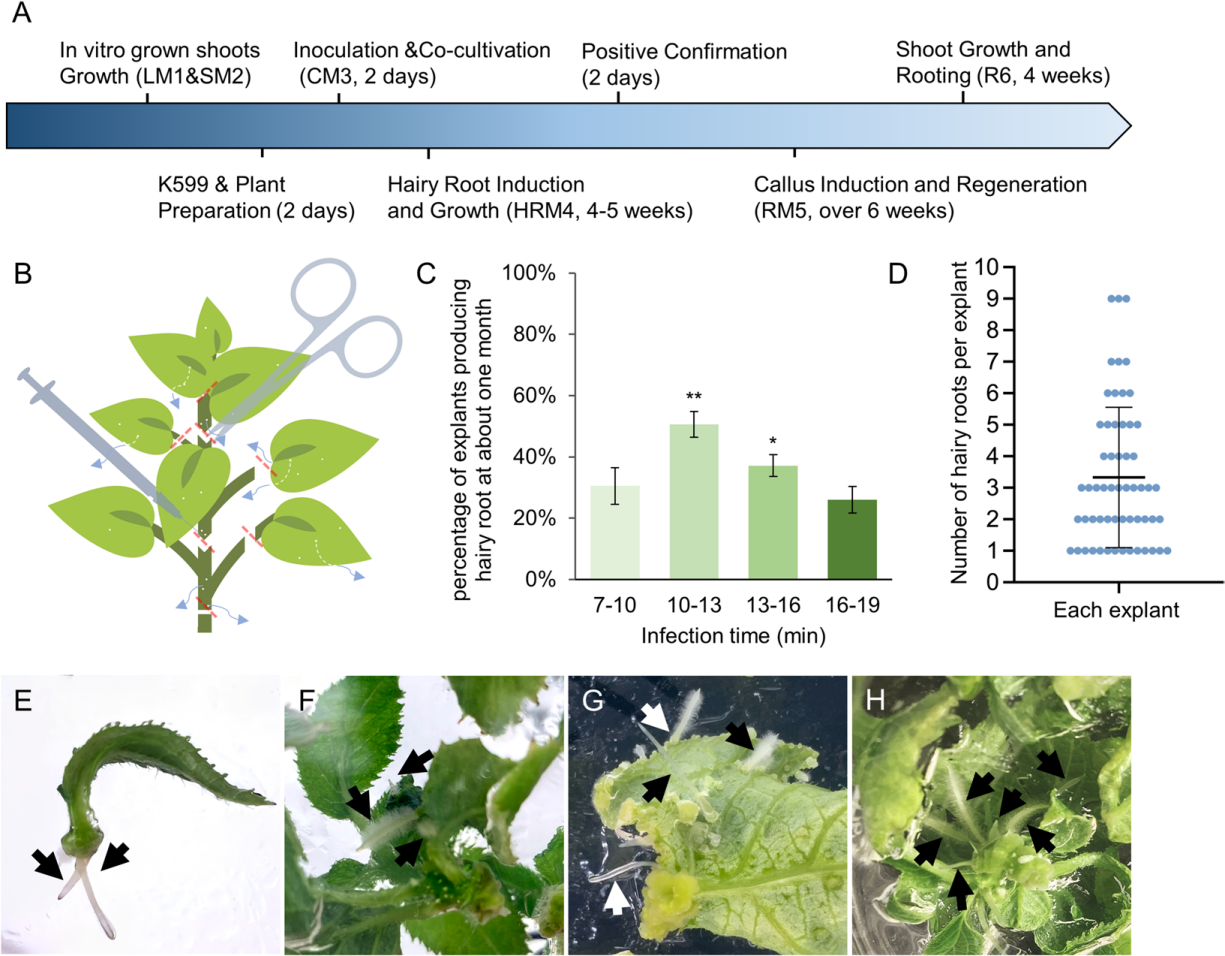
The hairy root induction of kiwifruit mediated by *Agrobacterium rhizogenes*. **A** Flow diagram of *Agrobacterium rhizogenes*-mediated transformation (ArMT) in kiwifruit. The Blue arrow represents the process direction of transformation, and the media component and information of each step are listed in Table 1. **B** The pretreatment of kiwifruit explants. The white dots represent the wounds caused at the leaves, veins, and young stems, and the red lines represent the position of the cut, the blue arrows indicate where the hairy roots may occur. **C** Effect of different infection times on induction efficiency. Significant differences compared with each other according to one-way ANOVA followed by Tukey’s multiple comparison tests are indicated with asterisks (*P* < 0.001). **D** The number of hairy roots produced on each explant is represented by blue dots. Mean ± SD was presented. **E**-**H** The hairy roots appeared in the wound of the petiole, leaves, leaf disc, and young stems, respectively

We used *A.rhizogenes* strain K599 to develop a fast and maker-free transformation method for kiwifruit, which usually takes about 4 months to obtain the transgenic plantlets (Fig. 1A). First, the young tissue culture plantlets are the best choices for hairy root induction. The plantlets were clipped at the petiole or hypocotyl to produce the explants (red lines in Fig. 1B). The explants were poked by a syringe needle to produce wounds, which is best on veins (Fig. 1B) when immersed in CM3 media (Table 1) with K599 containing pBI121-GUS or pCAMBIA1300-eGFP (Fig. S1). After 3-5 weeks, the induced hairy roots appeared from the section surface of petioles (Fig. 1E), the wounds of leaves (Fig. 1F and G), and young stems (Fig. 1H).

**Table 1:** Component of medium

Code	Medium Type	Cultivar	Additives
LM1	In vitro grown shoots	Ac^a^ & Ae^b^	MS^c^ supplemented with 0.6 mg/L NAA, 0.2 mg/L 6-BA
SM2	Shoot Growth	Ac	MS supplemented with 3.0 mg/ L zeatin, 0.1 mg/ L NAA, 30 g/ L sucrose
		Ae	MS supplemented with 2.0 mg/ L zeatin, 3.0 mg/ L 6-BA, 0.1 mg/ L IBA, 30 g/ L sucrose
CM3	co-cultivation	Ac & Ae	1/2 MS supplemented with 10 g/L sucrose, 100 μM Acetosyringone
HRM4-1	Hairy roots induction 1	Ac & Ae	MS supplemented with 25 g/L sucrose and 300 mg/L Cefotaxime^d^
HRM4-2	Hairy roots induction 2	Ac & Ae	1/2MS supplemented with 10 g/L sucrose and 300 mg/L Cefotaxime
RM5	Regeneration	Ac	SM2-c supplemented with 300 mg/L Cefotaxime
		Ae	SM2-e supplemented with 300 mg/L Cefotaxime
R6	Rooting		1/2 MS supplemented with 10 g/L sucrose, 0.8 mg/L IBA, 0.6% Agar

^a.^ Ac, *Actinidia chinensis* ‘Hongyang’

^b.^ Ae, *Actinidia eriantha* ‘White’

^c.^ MS, M519(Murashige &Skoog Basal Medium w/ Vitamins), PhytoTechnology

^d.^ Cefotaxime or Timentin

Based on the traditional genetic optimization scheme of kiwifruit mediated by *Agrobacterium tumefaciens*, the OD600 of the re-suspended bacterial solution was controlled between 0.6-0.8, and the infection time was controlled between 10 and 20 minutes to obtain a better transformation effect. The suspension of OD600 = 0.7 was evaluated for different infection times to obtain the best hairy root induction efficiency. Our results indicated that 10-13 min infection could obtain the highest induction rate that 50% of explants produced the hairy roots (Fig. 1C). Each explant could produce multiple hairy roots ranging from 1 to 9, averaging 3.3 (Fig. 1D).

**Fig. 2:**
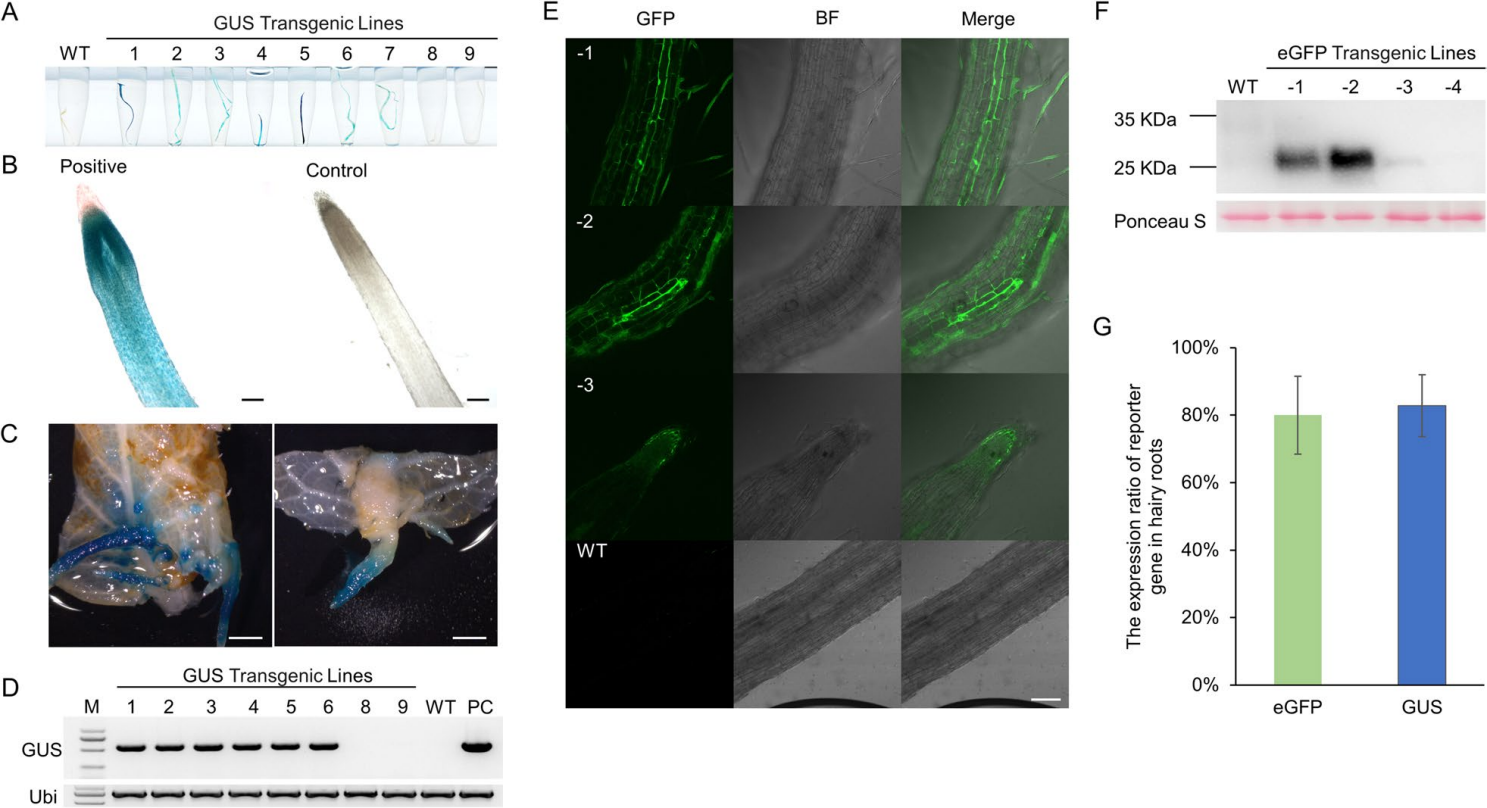
Characterization of transgenic roots of *GUS* and *eGFP*. **A**-**C** The GUS staining of nine 5 weeks transgenic hairy roots (**A**) and enlarged field of vision under optical microscope (**B**) and stereomicroscope (**C**) from *Actinidia chinensis* ‘Hongyang’. B. Bar = 2 mm. C. Bars = 200 μm. **D** Verification of T-DNA insertion fragment of *GUS* by PCR amplification. M, marker. WT, wild type. PC, positive control. **E** The eGFP fluorescence signal in 5 weeks transgenic hairy roots of *Actinidia eriantha* ‘White’. c, root tip. d, the wild type. Bars = 200 μm. **F** Western blotting with anti-GFP antibodies of 4 transgenic lines and wild type. Molecular mass markers are shown on the left. The expected size of *eGFP* is 26.9 KDa. **G** The expression ratio of *eGFP* and *GUS* gene in hairy roots. Data shown are averages ±SD; *n* > 15

To verify the transgenic hairy root of kiwifruit induced by *A.rhizogenes*, we first tested the system with two reporter genes, *GUS* and *eGFP* (Fig. 2). The expression cassettes of marker genes (*KanR* and *HygR*) were deleted from the constructs pBI121-GUS and pCAMBIA1300-eGFP, respectively, to generate maker-free transformation vectors (Fig. S1). *GUS* and *eGFP* vectors were used to transform *A. chinensis* ‘Hongyang’ and *A. eriantha* ‘White’, respectively. Based on the above-mentioned transformation method, the hairy roots were induced using the media without any antibiotics. For the pBI121-GUS vector, GUS staining assays of 9 independent GUS-transgenic hairy roots confirmed 7 lines of transgenic hairy roots showed the stable expression of *GUS* (Fig. 2A-C). The whole roots including root hairs were staining uniformly (Fig. S2). RT-PCR also confirmed the T-DNA insertion in the 7 transgenic lines (Fig. 2D). For the pC1300-eGFP vector, more than 20 independent lines were obtained and the GFP fluorescence was observed in 80% of transgenic hairy roots (Fig. 2E and G). Western blot with anti-GFP antibodies confirmed that *GFP* was stably expressed in the transgenic roots (Fig. 2F). These results indicated that K599-mediated marker-free transformation of hairy roots was successfully established in the kiwifruit, which is suitable for both *A. chinensis* ‘Hongyang’ and *A. eriantha* ‘White’.

**Fig. 3:**
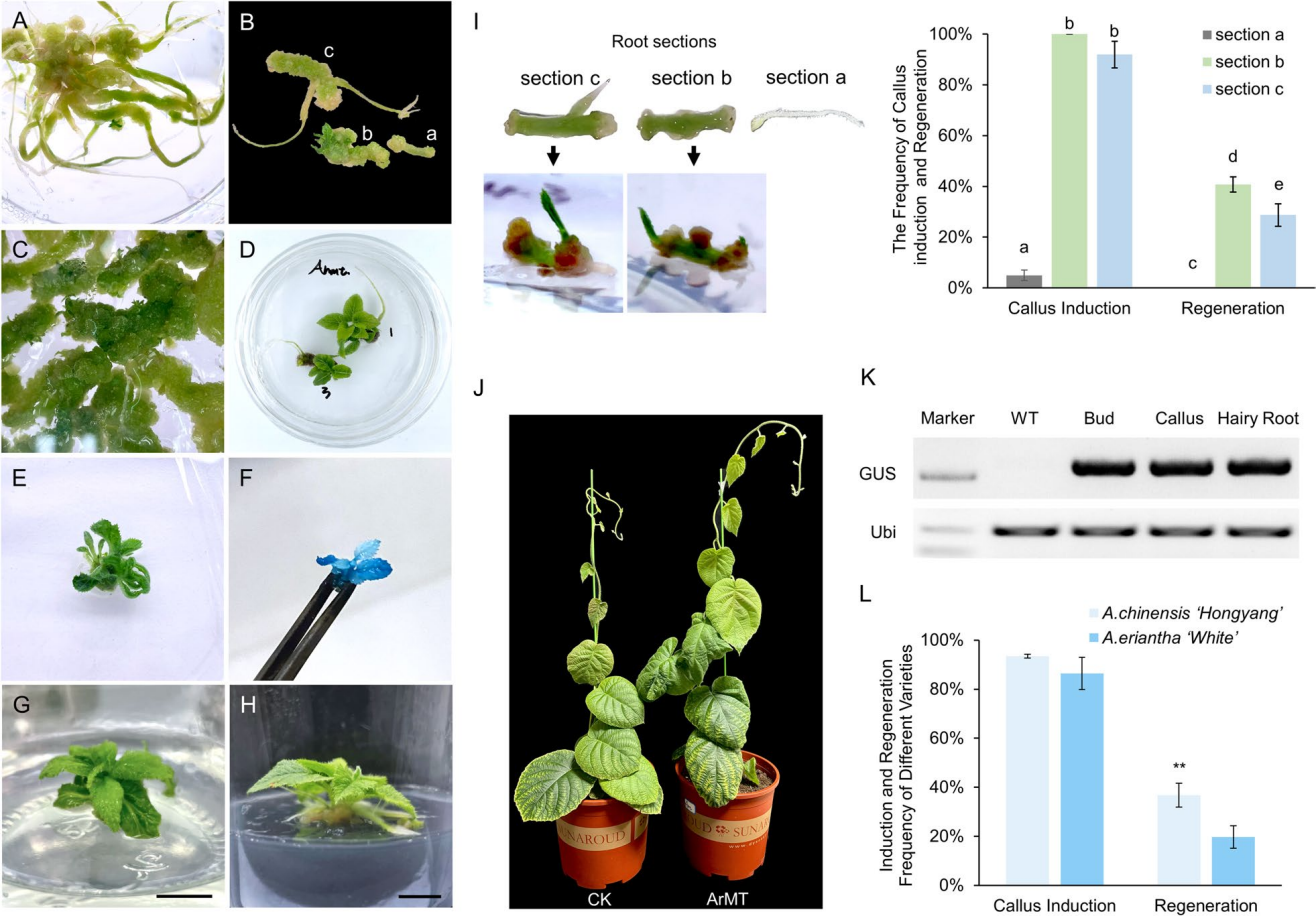
Callus induction and regeneration of hairy roots through a removing-root-tip method. **A** Callus induced in the presence and (**B**) removal of root tip; a, segment near the tip removing the root tip; b, mature zone; c, the zone with lateral roots. **C** Regenerative buds are produced during early induction on sections without the root tip. **D** The root segment of the root tip is removed to induce callus and regeneration. **E F K** GUS transgenic hairy root regeneration seedling and chemical staining, and GUS gene T-DNA insertion verification. **G H** Growth and rooting of regenerated seedlings induced by hairy roots. Bar = 1 cm. **I** The different sections of hairy root (section a, root tip; section b, the elongation zone; section c, the maturation zone with lateral roots) showed the different frequencies of callus induction and regeneration. **J** The WT and transgenic plants of 35S::GUS were grown in soil for 2 months. **L** Statistics of callus induction frequency and regeneration frequency from different varieties of kiwifruit hairy roots. Data shown are averages ±SD; *n* > 50 and asterisks indicated significant differences at *P* < 0.001

Based on previous studies, the induction of callus and shoot regeneration from hairy roots was achieved in various species (Choi et al. 2004). To establish callus derived from the root and shoot regeneration, the whole transgenic hairy roots of pBI121-GUS were transferred to the regeneration medium (RM5) containing high concentrations of cytokinin but low auxin (Table 1). After being cultured for about 4 to 5 weeks, the calluses were successfully induced from hairy roots, but the shoot regeneration is very difficult and with very low efficiency (Fig. 3A and J). Occasionally, we found that it could increase the regeneration efficiency from 10 to 37% by removing root tips (Fig. 3B, C, and J). The apical tissue near the root cap (about 0.8 cm) seemed to be less likely to induce callus and shoot, as indicated by ‘a’ in Fig. 3B. The elongation zone of root (b in Fig. 3B) is the best part for inducing callus and regeneration (Fig. 3I). The zone with lateral roots (c in Fig. 3B) was easy to form callus but less efficient to induce regeneration (Fig. 3I).

Without primary and lateral root tips, the sections of root elongation and mature zones are capable of producing calluses and regenerating shoots effectively (Fig. 3C-E, I). Callus induction and shoot regeneration were performed on the media without any selective pressure for plants. GUS staining indicated that *GUS* gene was stably expressed in the entire shoots generated from the transgenic hairy roots (Fig. 3F). PCR assays also verified the T-DNA insertion in the callus and shoot (Fig. 3K). After growing on R6 medium for about 3 weeks, all the regenerated shoots were rooted. As previously described, some regenerated plants from hairy roots may have wrinkled leaves, extremely abundant and oblique roots, reduced apical dominance, and reduced internode length and leaf size (Hu and Du 2006). In our study, however, the rooting and growth of transgenic shoots were not much different from that of wild-type plants (Fig. 3G, H, and J). These results indicated that our removing-root-tip method can effectively induce the callus and shoot regeneration and the exogenous DNA was inserted in the genome and stably inherited by using our maker-free transformation system.

In addition, the statistical comparison showed that the regeneration probability of *Actinidia eriantha* ‘White’ was lower than that of *Actinidia chinensis* ‘Hongyang’ (Fig. 3L). Apparently, it requires further investigations to reveal underlying mechanisms.

### *A.Rhizogenes*-mediated gene editing of *AcCEN4* and *AeCBL3*

**Fig. 4:**
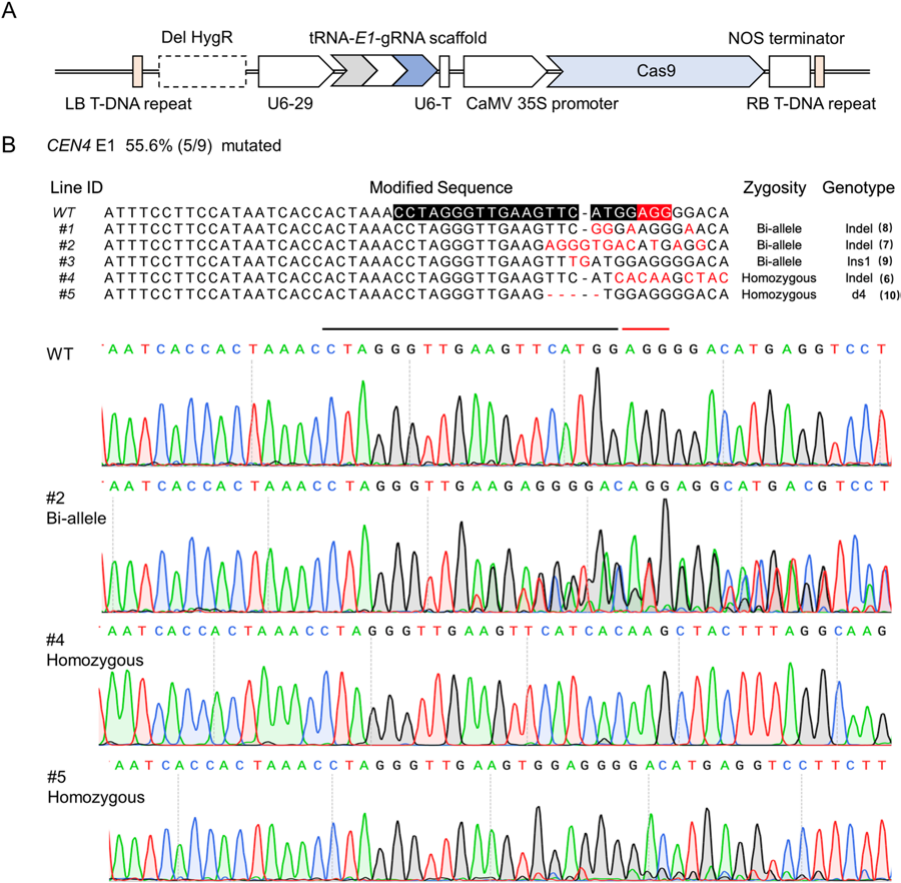
*Agrobacterium rhizogenes*-mediated gene editing of *CEN4* gene in kiwifruit. **A** Polycistronic tRNA-gRNA (PTG)/Cas9 vector structure targeting the *CEN4*-E1 site, the screening marker gene was removed and represented by a black dotted box, the gray arrow, white arrow and blue arrow in the PTG structure represent tRNA, sgRNA and gRNA scaffold, respectively. **B** Edited results of *CEN4* gene target region sequences in 9 transgenic hairy roots, of which 5 were detected editing. Black and red indicate the target sequence and PAM region. Indel, small fragment insertion and deletion variation; Ins, insertion; d, deletion; WT, wild type. The number in brackets indicates the clones of PCR products for Sanger sequencing

While the CRISPR/Cas9 system has been applied in multiple plant species including kiwifruit, it has not been evaluated with *A.rhizogenes*-mediated transformation in kiwifruit. To determine the efficiency of *A.rhizogenes*-mediated gene editing in kiwifruit, we used *CEN4* as a target gene, which has been successfully edited in the previous study (Varkonyi-Gasic et al. 2019). The gRNA E1 target sequence of *CEN4* gene was obtained from the previous study that successfully performed *CEN4* gene edition (Varkonyi-Gasic et al. 2019). The pCAMBIA1300 vector was digested to remove the *HygR* marker gene as a marker-free backbone to construct an optimized Polycistronic tRNA-gRNA (PTG)/Cas9 system (Xie et al. 2015), which we designated as U6-tRNA-CEN4E1-gRNA-CaMV35s-Cas9/pCAMBIA1300 (Fig. 4A). After transforming *Actinidia chinensis* ‘Hongyang’ through our *A.rhizogenes*-mediated method, we obtained 9 independent transgenic lines of hairy roots. The PCR and Sanger sequencing indicated *AcCEN4* was successfully edited in 5 of 9 lines and the edit efficiency is close to 55.6% (Fig. 4B). PCR products were cloned to the pESI-Blunt vector and at least 10 clones from each line were subjected to Sanger sequencing. The results indicated that the 5 edited lines showed different types of editing including deletion, insertion, and the combination of both (Fig. 4B). Surprisingly, 2 of 5 edited lines are homozygous (Fig. 4B). These results indicated that *A.rhizogenes*-mediated CRISPR/Cas9 system is highly efficient.

**Fig. 5:**
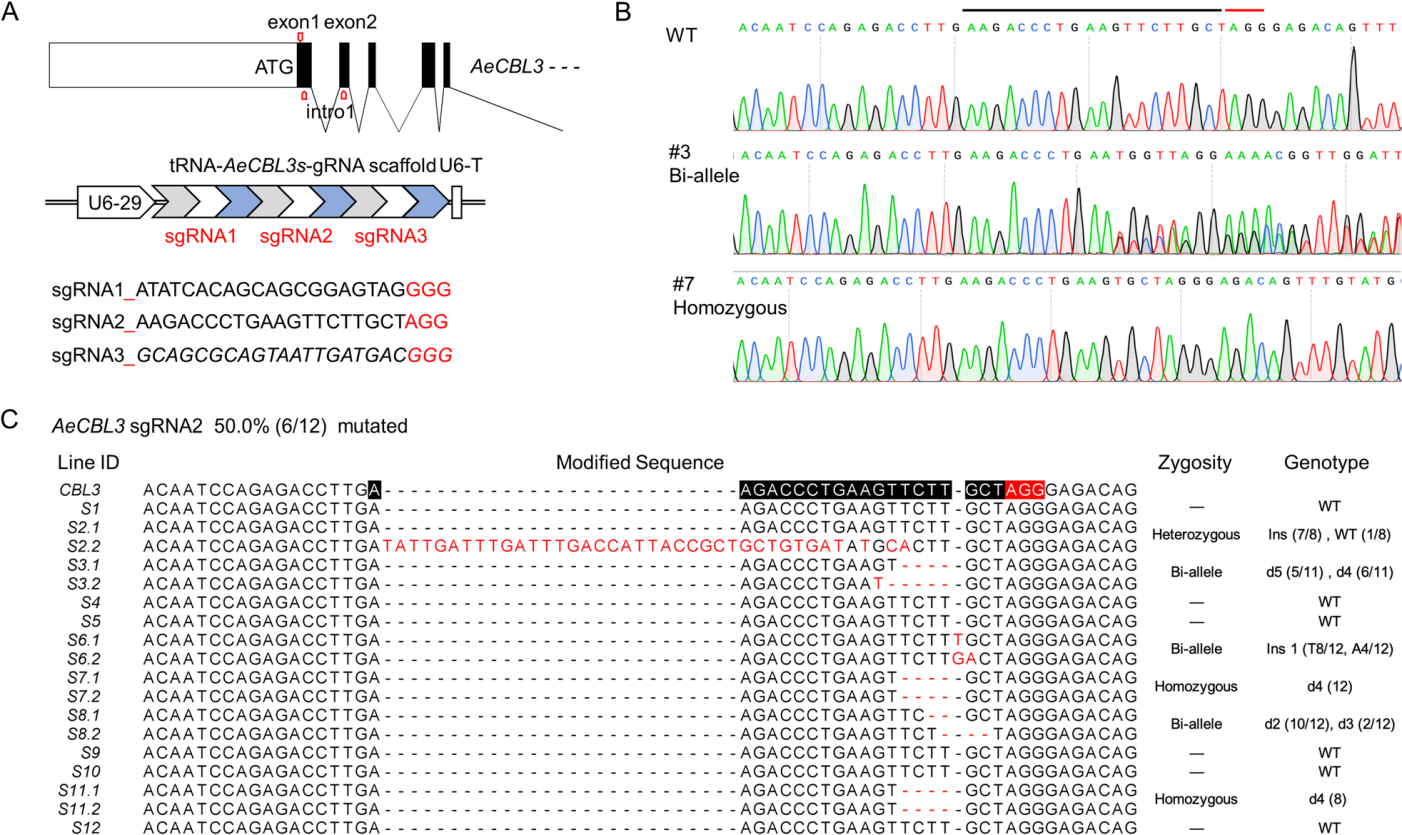
The gRNA screening and gene editing of *AeCBL3*. **A** Design of 3 targeted sequences on the first exon and the second exon of the *AeCBL3* gene represented by red arrows. The gray arrow, white arrow, and blue arrow in the PTG structure represent tRNA, sgRNA, and gRNA scaffold, respectively. The multiple editing structure is composed of three tRNA-gRNA combinations. **B** Genome sequence of the sgRNA2 site region in the transgenic hairy root, the black and red lines represent the targeted sequence and the PAM region. **C** Gene modification of 12 independent transformed hairy roots, and genotype classification. Monoclonal sequencing of each independently transformed hairy root was performed to analyze specific gene editing forms, all the modified sequences were displayed, while the genome sequences of other lines were not changed. Target sequences and PAM regions were labeled with black and red lines. Ins, insertion; d, deletion; WT, wild type. The number in brackets (a/b), a represents the number of colonies of this type, b represents the total number of colonies

In the model plants such as Arabidopsis and rice, protoplasts were used to screen effective gRNA. However, the protoplast isolation is very difficult in kiwifruit. The fast screen system of effective gRNA is lacking in kiwifruit. To check whether *A.rhizogenes*-mediated transformation is suitable for gRNA screening, we tried to edit a *Calcineurin B-like 3* gene (DTZ79_17g06240) of *A. eriantha* ‘White’ (*AeCBL3*). First, the full-length cDNA and the genomic sequence of *AeCBL3* were amplified and aligned to ensure the accuracy of exons for sgRNA design. Three sgRNAs targeting *AeCBL3* (sgRNA1-3) were designed according to the website (http://crispor.tefor.net/) and ligated using tandemly arrayed tRNA-gRNA structure as described in a previous study (Xie et al. 2015) (Fig. 5A). Through *A.rhizogenes*-mediated transformation of ‘White’, we obtained 12 transgenic lines (S1-S12) of hairy roots in 3 weeks. PCR and sequencing indicated that only *sgRNA2* successfully induced gene editing of *AeCBL3* (Fig. 5B). For the target sequence of *sgRNA2*, gene editing was observed in 6 of 12 lines (Fig. 5C). The three lines (S3, S6, and S8) showed the different modifications in two chromosomes (Bi-allele in Fig. 5C) and two lines (S7 and S11) are homozygous at the editing site (Fig. 5C). These results indicated that *A.rhizogenes*-mediated CRISPR/Cas9 system is suitable for screening effective gRNA and performing gene editing efficiently in kiwifruit.

### *AeCBL3* mediates the formation of calcium oxalate crystals

**Fig. 6:**
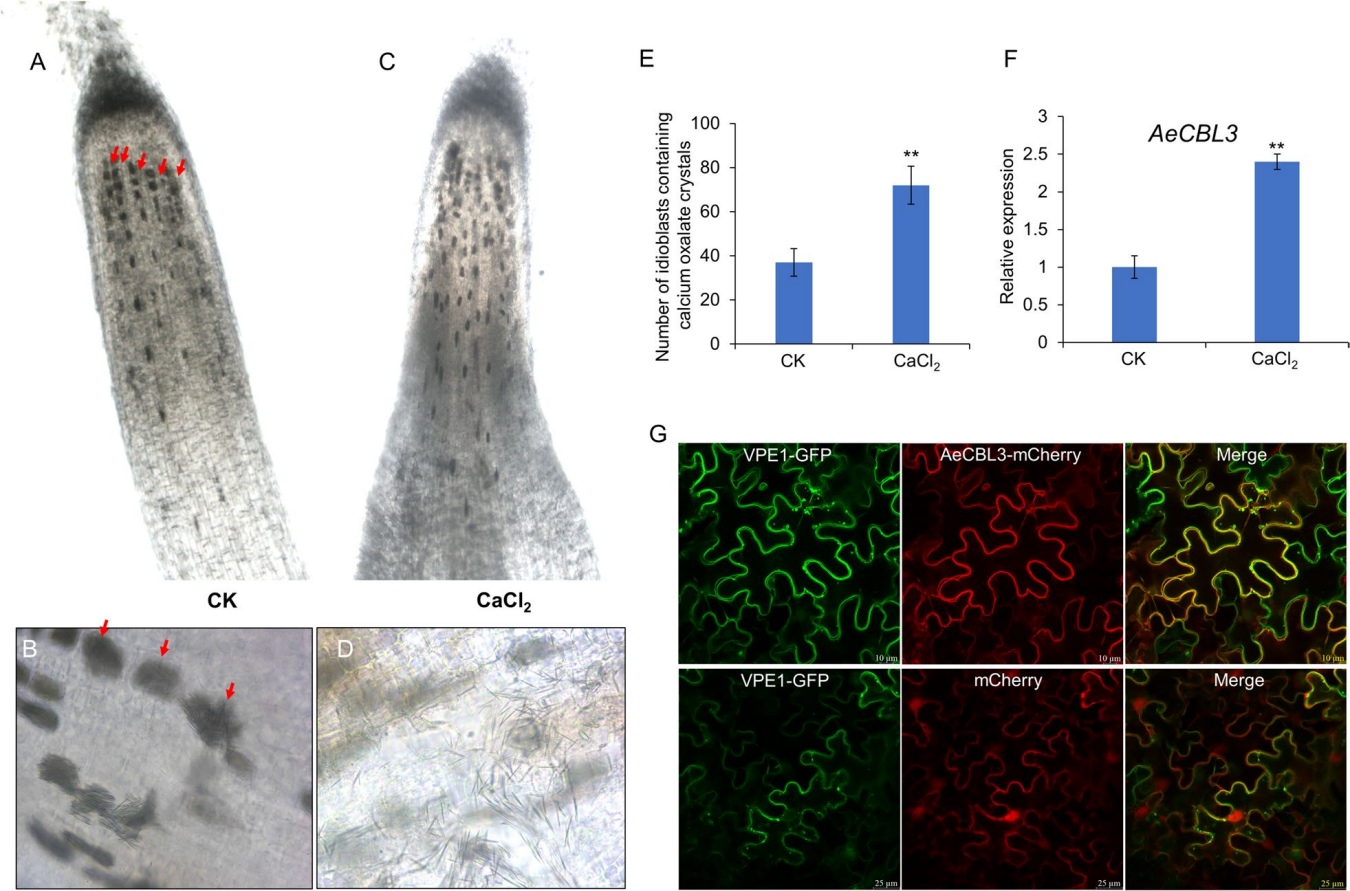
CaCl_2_ treatment increased the formation of calcium oxalate crystals in the root tip. **A**-**D** Microscopy observation of primary root of ‘White’ treated without (**A**) and with 15 mM CaCl_2_ (**C**) for 1 week. Red arrows indicated the crystal idioblasts. The bundled needle-shaped raphide crystals in the idioblasts (**B**) and the released raphide crystals from broken idioblasts (**D**). **E** The counted number of crystal idioblasts in root tips of ‘White’ treated without (CK) and with CaCl_2_ Data shown are averages ±SD; *n* > 10. ‘**’ indicates significant differences at *P* < 0.001. **F** qRT-PCR analysis of AeCBL3 in the roots treated without (CK) or with CaCl_2_. The three independent experiments were performed and three biological replicates for each treatment. ‘**’ indicates significant differences at *P* < 0.001. **G** Confocal microscope observation of AeCBL3-mCherry, mCherry, and vacuolar membrane marker protein VPE1-GFP transiently expressed in the tobacco leaves

Previous studies showed that kiwifruit accumulates raphide crystals of calcium oxalate in the fruit (Perera et al. 1990; Nguyen and Savage 2013). In this study, we observed that the raphide crystals also accumulated in the root, especially the root tip of *A. eriantha* ‘White’ (Fig. S3). CaOx crystals are deposited in the vacuole of the cell, which is referred to as crystal idioblast (Franceschi and Nakata 2005). As indicated by red arrows in Fig. 6A, crystal idioblasts were easily found in the root tips. The needle-shaped raphide crystals were observed as bundles of hundreds in the idioblasts (Fig. 6B) and the needle-shaped crystals could be released when idioblast cells were broken (Fig. 6D). Besides, the exogenous application of 15 mM CaCl_2_ significantly increased the number of idioblasts in the root tips (Fig. 6D and E). Our qRT-PCR assays showed that the CaCl_2_ treatment enhanced the expression of *AeCBL3* (Fig. 6F). Subcellular localization analysis showed that AeCBL3-mCherry is associated with the vacuolar membrane, as indicated by the overlap of mCherry signal with green fluorescence signal of VPE1-GFP (Fig. 6G), a marker protein of vacuolar membrane (Xu et al. 2019).

**Fig. 7:**
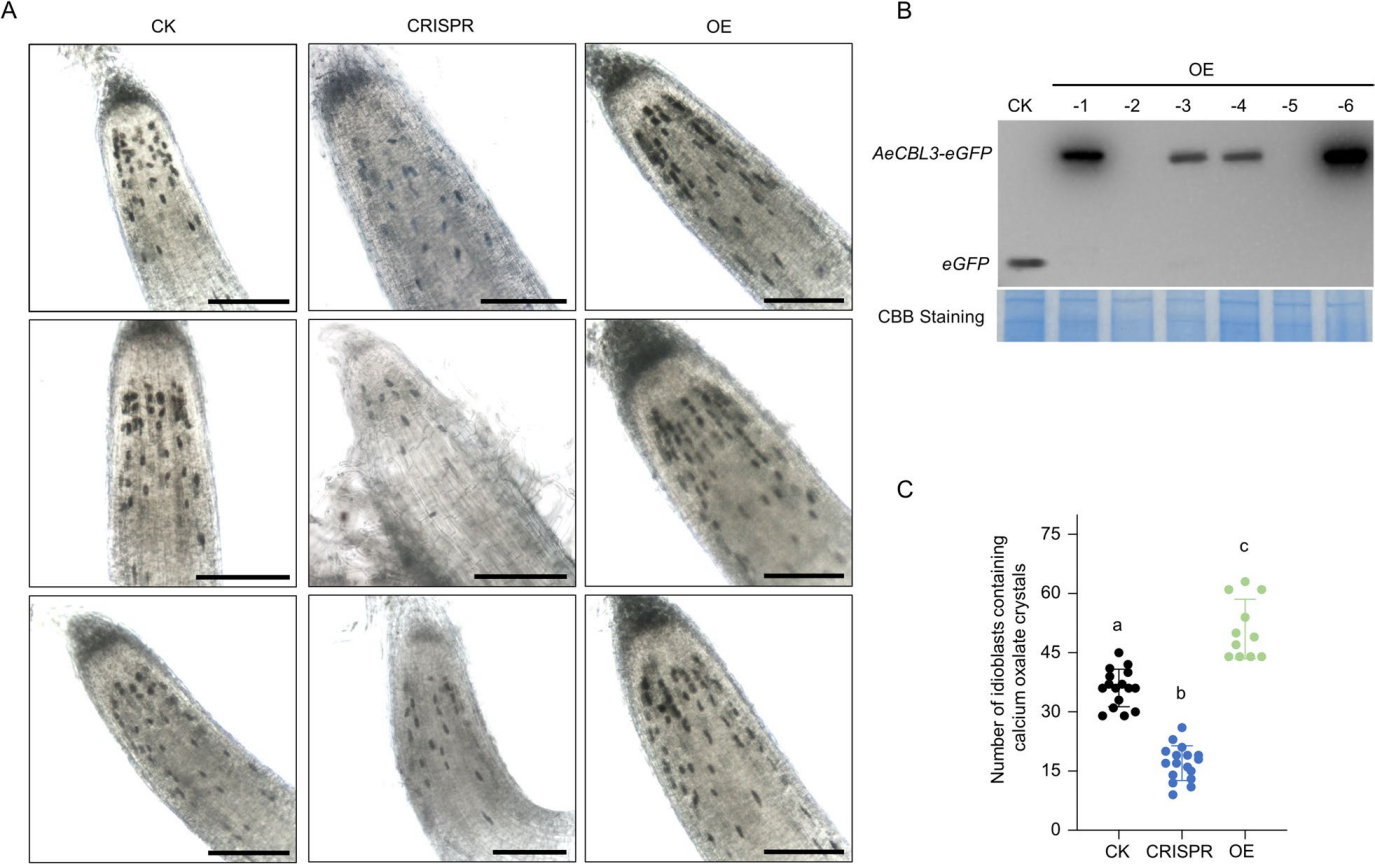
*AeCBL3* mediates the formation of calcium oxalate crystal idioblasts in kiwifruit. **A** Observation of idioblasts in hairy roots under an optical microscope. CK, Control lines; CRISPR, *AeCBL3*-edited lines; OE, Overexpression lines*.* Bar = 200 μm. **B** Western blot analysis of CK and OE lines using anti-GFP antibodies. **C** The number of idioblasts containing calcium oxalate crystals in the independent lines of Control, Overexpression, and CRISPR was counted, which was represented by black dots, green dots, and blue dots, respectively. Data shown are averages ±SD; *n* > 10. Significant differences compared with each other according to two-way ANOVA followed by Tukey’s multiple comparison tests are indicated with a, b, and c (*P* < 0.001)

To assess whether *AeCBL3* mediates the formation of calcium oxalate crystals, we knocked out *AeCBL3* through *A.rhizogenes*-mediated CRISPR/Cas9 system as the above-mentioned descriptions (Fig. 5A, Fig. S1C). We observed the homozygous and bi-allele lines of *AeCBL3*-edited roots (CRISPR in Fig. 7A) and found that the knockout of *AeCBL3* significantly reduced the number of crystal idioblasts in root tips (Fig. 7C). Besides, the observation of CaOx crystals using polarized light on confocal microscope also indicated that overexpression of *AeCBL3* increased but CRISPR-mediated knockout decreased the accumulation of CaOx crystals in the leaves of transgenic lines (Fig. S3). These results indicated that *AeCBL3* is required for the formation of CaOx crystals and idioblasts in kiwifruit.

Meanwhile, the CDS of *AeCBL3* was fused with *eGFP* and inserted in the vector pCAMBIA1300 to construct overexpression (OE in Fig. 7A) vector p1300-AeCBL3-eGFP. The empty vector p1300-eGFP was taken as control (CK in Fig. 7A). Both vectors were used to transform *A. eriantha* ‘White’ by *A.rhizogenes*-mediated method. Western blot using anti-GFP antibodies indicated that eGFP and AeCBL3-eGFP were substantially expressed in the transgenic roots of CK and OE lines, respectively (Fig. 7B). The transgenic roots (Fig. 7A and C) and leaves (Fig. S3) of OE lines showed significantly more idioblasts than that of CK lines. These results indicate that *AeCBL3* overexpression can increase the formation of CaOx crystals and idioblasts.

## Discussion

The *A.tumefaciens*-mediated transformation of kiwifruit is a time-consuming process involving extremely tedious experimental procedures, months of cycles, and effective stress screening. The *A.tumefaciens*-mediated transformation occurs in a few infected plant cells at a very low frequency. Therefore, the selection marker genes are indispensable for screening the rare plant cells that have taken up foreign DNA (de Vetten et al. 2003). The public concerns about food safety arise from the usage of antibiotic- and herbicide-resistance genes. The marker-free transformation contributes to the public acceptance of transgenic crops. So far, the marker-free transformation of kiwifruit has not been established yet. In this study, we developed an *A.rhizogenes*-mediated marker-free method that shortens the transformation process to about 4 months (Fig. 1). Conveniently, most plant materials, such as hypocotyls, leaves, and petioles, can be used to induce hairy roots (Fig. 1). And it will be induced within a short period, which varies from 1 week to over a month depending on different plant species. As a “natural genetic engineer”, the Ri plasmid of *A. rhizogenes* transforms the plant cells by introducing its T-DNA into the genome of plant cells, and the transformed plant cells grow in the form of hairy roots (Chilton et al. 1982; Christey 2001), which means that the exogenous expression cassette can be transferred into the plant genome and eliminates the need for hormones and screening pressure (Veena and Taylor 2007). Indeed, our results indicated that the transgenic roots of kiwifruit were successfully obtained by using our method without any antibiotics (Fig. 2). Besides, the efficiency of our method is very high, as indicated by the results that about 80% of induced hairy roots contained the insertion of foreign DNA and showed the expression of foreign genes (*eGFP* and *GUS*) (Fig. 2). Our marker-free transformation is suitable for both *A.chinensis* ‘Hongyang’ and *A.eriantha* ‘White’. Further investigations are needed to determine whether other *Actinidia* species can be transformed by using this method.

Another problem of the *A.tumefaciens*-mediated transformation is the possibility of chimera (Chen 2011), which arises because the transformed and non-transformed cells together develop into a single chimeric transgenic plant. The chimeric tissues will bring a lot of troubles to genotyping analysis, especially for gene editing. For *A. rhizogenes*-mediated transformation, the previous studies indicated that the transformed root from single root tip was shown to be a cellular clone (Chilton et al. 1982; Chen et al. 2018). In this study, the results of *A. rhizogenes*-mediated gene editing showed that there are a couple of homozygous knockout lines for both *CEN4* and *AeCBL3* genes (Figs. 4 and 5), indicating that the transgenic root indeed developed from a single infected cell. Besides, the transgenic roots and regenerated shoots of *35S-GUS* showed uniform GUS staining (Fig. 2B and 3F). These results indicated that there is no chimera problem for *A. rhizogenes*-mediated transformation and gene editing of kiwifruit.

In the model plants such as Arabidopsis and rice, protoplasts were used to screen effective gRNA. However, protoplast isolation and transformation are very difficult in kiwifruit (Oliveira and Pais 1991). So far, we lack the fast screen system of effective gRNA in kiwifruit. Our results indicate that we can screen out one effective gRNA for *AeCBL3* in 3 weeks (Fig. 5). Moreover, our method provides higher efficiency of gene editing. The previous study obtained 6 gene-edited plants of *CEN4* gene from more than 20 independent transgenic lines (Varkonyi-Gasic et al. 2019). In our study, we obtained 5 gene-edited roots of *CEN4* from 9 transgenic roots (Fig. 4B). Moreover, we obtained 6 gene-edited lines from 12 transgenic roots for *AeCBL3* gene (Fig. 5C). For both genes, the editing efficiency is about 50%. Besides, we obtained several homozygous lines for both genes. These results indicated our method provided a fast and convenient way to screen effective gRNA for target genes and established a highly efficient system for gene editing of kiwifruit.

Previous studies have shown that *A. rhizogenes* NIASE1724 and ArM123 may also apply to kiwifruit *A. deliciosa A. Chev.* (Yazawa et al. 1995; Yamakawa and Chen 1996) and their efficiency of hairy root induction ranged from 2.5 to 30%, respectively. In this study, we used a different strain K599 to infect ‘Hongyang’ and revealed that about 50% of explants produced hairy roots and each explant could generate an average of 3.3 hairy roots (Fig. 1). Approximately 80% of hairy roots are transgenic (Fig. 2). Besides, we created a removing-root-tip method to solve the regeneration problem. Through our removing-root-tip method, the regeneration efficiency of hairy roots was increased from 10% to about 37% (Fig. 3J). Although the underlying mechanisms remain unknown, we think it might be a common phenomenon and could be applied to other plant species. Similarly, a recent study (Cao et al. 2023) developed the cut-dip-budding delivery system using *A. rhizogenes*, which includes a step of cutting the transgenic root into a few segments to induce regeneration. Therefore, it will be interesting to investigate why the removing-root-tip step increases the regeneration efficiency in the future.

CaOx crystals are widely present in most photosynthetic organisms and commonly produced in the vacuoles of specialized cells called crystal idioblasts. Their speculated functions include excess calcium excretion, heavy metal/oxalate detoxification, light reflectance, and protection against grazing herbivory (Franceschi and Nakata 2005). CaOx crystals occur in a wide variety of morphologies including block-like rhombohedral, large elongate rectangular styloids, needle-shaped raphide, crystal sands, and druses (Franceschi and Nakata 2005). Kiwifruit accumulates needle-shape raphide crystals in fruits, which are responsible for mouth irritation or catch when eating kiwifruit (Perera et al. 1990). Therefore, the reduction of raphide crystal accumulation in kiwifruit will improve the fruit texture and safety. In our study, we found that kiwifruit root tips accumulate massive raphide crystals and the exogenous CaCl_2_ treatment significantly increased the number of idioblasts in the root tip (Fig. 6), suggesting that kiwifruit used the CaOx formation to excrete excess calcium and to maintain calcium homeostasis. Although CaOx crystals have been discovered for several centuries, it remains largely unknown how CaOx crystals are generated. CaOx crystals are composed of calcium and endogenously synthesized oxalic acid. An Acyl Activating Enyzme 3 (AAE3) catalyzes the key step in a major pathway of oxalate degradation in plants (Foster et al. 2012). Knocking down *Medicago truncatula AAE3* (*MtAAE3*) led to a reduction in oxalate degradation and overaccumulation of druse CaOx crystals in Medicago (Foster et al. 2016). Biochemistry methods indicated calcium channels are enriched in the idioblast of *Pistia stratiotes L*. and possibly involved in calcium oxalate crystal formation (Volk et al. 2004). However, no specific gene in calcium signal has been genetically demonstrated to regulate the CaOx crystal formation.

In our study, we revealed that *AeCBL3* is upregulated by CaCl_2_ treatment (Fig. 6). CaOx crystals are accumulated in the vacuole and AeCBL3 is localized on the vacuolar membrane (Fig. 6G). Overexpression of *AeCBL3* in *A. eriantha* ‘White’ remarkably enhanced the accumulation of crystal idioblasts in root tips (Fig. 7) and leaves (Fig. S3). The knockout of *AeCBL3* using *A.rhizogenes*-mediated CRISPR-Cas9 system significantly decreased the accumulation of crystal idioblasts in root tips (Fig. 7) and leaves (Fig. S3). These results indicated that *AeCBL3* mediates the formation of CaOx crystal and idioblast in kiwifruit. Our work provides a candidate gene for genetic manipulation of reducing CaOx formation and improving fruit texture and safety in kiwifruit. In plants, the CBL protein family functions as calcium sensors that percept calcium signals by interactions with a group of CBL-interacting protein kinases (CIPKs). The CBL-CIPK complexes play a key role in response to extracellular cues such as abiotic stresses and nutrient deprivation (Tang et al. 2020). In Arabidopsis, the vacuolar membrane-localized CBL2/3 interacts with CIPK3/9/23/26 to mediate potassium (K^+^), magnesium (Mg^2+^), and manganese (Mn^2+^) homeostasis by regulating V-ATPase and ion transporters (Tang et al. 2012; Ju et al. 2022; Tang et al. 2022). Therefore, we speculate that AeCBL3 might interact with some CIPKs to regulate the activity of vacuolar calcium transporters and thereby mediate CaOx crystal formation in the vacuole of kiwifruit. Apparently, it requires further investigations to prove the speculation.

Together, our work developed a fast and convenient maker-free transformation and highly efficient CRISPR-Cas9 gene editing system for kiwifruit. Meanwhile, we demonstrated that AeCBL3 mediates the formation of CaOx crystal and idioblast in kiwifruit, which provides a novel clue to elaborate the mechanisms of CaOx crystal formation.

## Materials and methods

### Plant materials and tissue culture

All leaves and vines of kiwifruit (*Actinidia chinensis* ‘Hongyang’ and *Actinidia eriantha* ‘White’) were collected from the Botanical Experimental Garden of Anhui Agricultural University in Hefei, Anhui Province. The collected plant tissue samples were sequentially placed in 70% ethanol for 2 min, then sterilized with 1% NaClO for 2 min. The sterilized plant samples were rinsed with sterile water and blotted dry the water on sterile filter paper before planting on growth media.

To initiate new buds and establish axenic bud culture, vines were cut into ∼7 cm lengths (with at least one node remaining) and about 2 cm of the cane was immersed into LM1 media supplemented with Plant Preservative Mixture (Coolaber PTC1000). After 4 ~ 5 weeks, newly initiated leaves excised from in vitro grown canes were cut into ∼10 × 10 mm leaf strips for regeneration experiments or regenerated on callus induction and regeneration medium (SM2) to regenerate sterile hypocotyls for transformations. All tissue culture conditions were at 24 °C ± 2, 16/8 h photoperiod. The pH of the various media was adjusted to 5.8 before autoclaving at 121 °C for 20 min.

### Construct modification of marker-free vector and CRISPR vector

Since the convenience of *A.rhizogenes* transformation does not require the participation of screening pressure, Fragments containing the expression cassettes were ligated into the plant transformation vector deleting Marker cassettes, generating pCAMBIA1300-EGFP (marker-free) and pBI121-GUS (marker-free) (Fig. S1A and Fig. S1B). The *HygR* gene and CaMV35S promoter (enhanced) in the T-DNA region of the pCAMBIA1300 vector were replaced by a short sequence, which is a synthesized DNA fragment containing multiple clone sites that can be used for insertion of another gene or expression unit in the future. Then, the *eGFP* expression unit was introduced in this marker-free background. The *NeoR/KanR* marker expression cassette in the pBI121 vector was deleted in the same way. Moreover, the CRISPR drive could also allow the development of a marker-free resistant strategy with modifications on the drive cassette.

Kiwifruit genomic DNA was extracted from leaves of Actinidia eriantha ‘White’. The genomic DNA extraction was performed with the cetyltrimethylammonium bromide (CTAB) method (Murray and Thompson 1980). To design the sgRNA sequence targeting the *AeCBL3* gene, the first two exons and intron sequences were amplified from ‘White’ genomic DNA, and cloned into the pESI-Blunt simple vector (Yeasen Bio) for sequencing. All targeting sequences were designed with the sgRNA designer of guide-design-resources (https://zlab.squarespace.com/guide-design-resources). Then the complete PTG structure of U6-29-tRNA-sgRNA containing three targeting sequences was synthesized through gene synthesis service (Generalbiol). The PTG structure was excised by restriction enzymes and inserted upstream of the Cas9 gene in the pCAMBIA1300-Cas9 vector via the KpnI/BamHI restriction sites with T4 DNA Ligase. All fast Endonucleases were purchased from Monad Biotech.

### Agrobacterium strains preparation

The *A. rhizogenes* strain K599 (Tolobio) was used in this study. The vectors used in the study were all endowed with a Kanamycin selective marker. Briefly, all vectors were transformed into *A. rhizogenes* K599, and a single positive colony was inoculated and cultured in 1 ml of YEB liquid medium (5 g/L beef extract, 0.5 g/L MgCl_2_, 1 g/L yeast extract, 5 g/L peptone, 5 g/L sucrose) supplemented with 50 mg/L strep and 50 mg/L Kana at 28 °C, 200 rpm for primary culture. Then 1 ml bacterial liquid was inoculated in YEB liquid media and incubated overnight to a final optical density at 600 nm (OD600) of 1.0. The overnight culture was centrifuged to remove the YEB medium and re-suspended in 1/2 MS liquid media (pH = 5.6) supplemented with 100 μM Acetosyringone to OD600 of 0.6 to 0.8, then set to shake slowly at 28 °C for 30 min.

### Transformation procedures

The part tissues of hypocotyl with leaf and petiole excised from sterile tissue culture plantlets regenerated in vitro (grown on SM2 medium for about 4 weeks) were completely immersed and gently shaken with the bacterial solution of K599 for 10-12 min, then blotted dry with a sterile filter paper and transferred to a co-cultivation medium (CM3) in the dark. Furthermore, wounds caused by syringe needles of scissors or syringes given to transformed plant tissues may contribute to the induction of more roots. After 2 days of dark co-cultivation, the plant tissue samples were transferred to MS media supplemented with 300 mg/L cefotaxime sodium (HRM4) to induce the hairy roots (Table 1, Fig. 1). In early induction, the culture can be transferred to the same new medium multiple times if *A. rhizogenes* can’t be effectively inhibited. The culture medium at this stage does not require any hormones. The root tip (approximately 1 cm) of hairy root was cut off. The root tip is used for identification and detection, which is difficult to generate callus. The latter was cut into multiple pieces and placed on RM5 medium for callus induction. For shoot regeneration, the induced callus was cultured on the same RM5 medium for more than 1 month and shoots would automatically develop from the callus. The media should be constantly changed (usually every 2 weeks).

### GUS staining and GFP fluorescence signal

To confirm the histological expression pattern of GUS driven by the different promoters histochemical staining was performed as described previously (Zhuang et al. 2021). The leaves, hairy roots of transgenic kiwifruit plantlets, and fruit slices were immersed in the staining buffer under vacuum for 30 min and then incubated overnight at 37 °C. Chlorophyll was removed by incubating stained leaf tissue in 70% ethanol for 6 h, then 80% ethanol for 6 h, and 100% ethanol for 12 h. The enlarged field of view was observed using an optical microscope (Nikon Eclipse E200) and a stereomicroscope (ZEISS Stemi 508). The eGFP fluorescence signals of hairy roots were observed and imaged with a confocal laser-scanning microscope (Olympus FV1000).

### Western blot analysis

Plant samples were weighed and frozen in liquid N_2_, and ground in four volumes of 2 × loading sample buffer (100 mM Tris-HCl(pH = 6.8), 4%SDS, 0.2%BPB, 20%Glycerol, 5%β-Me). Total proteins were separated by SDS-PAGE, transferred to a PVDF (0.45 mm) blotting membrane, and probed. Monoclonal antibodies against GFP (300943) and HRP-conjugated goat anti-rabibit secondary antibodies (ZB-5301) were purchased from ZEN BIO and ZSGB-BIO, respectively.

### Genotyping analysis and sanger sequencing

Genomic DNA was extracted from hairy root lines using the one-step method of DNA extraction. Significantly, the presence of SDS components in the one-step extract had inhibitory effects on the PCR reaction. To reduce the inhibition, 1 μL DNA extract solution was added to the total 50 μL PCR reaction system as the template for amplification. First, PCR products were subjected to Sanger sequencing. If the sequence is heterozygous, the PCR products were inserted into the pESI-Blunt vector and converted the vector into the DH5α cell for selection and sequencing. The PCR products obtained directly from each transgenic line and the monoclonals (*n* > 10) ligated to the cloning vector were subjected to Sanger sequencing to determine whether the DNA fragment was edited. Sanger sequencing was performed by TsingKe Biotech. The alignment analysis was performed using Jalview and Snap Gene software.

### The observation of calcium oxalate crystal

To observe the accumulation of calcium oxalate crystals, root and leaf tissues were trimmed to a suitable size, and then visualized using an optical microscope (Nikon Eclipse E200) and polarized light of a confocal laser-scanning microscope (Olympus FV1000), respectively. The number of CaOx crystals in roots under bright field and the area of CaOx crystals observed by polarized light were statistically and quantitatively analyzed using ImageJ.

### Statistical analysis

Statistical tests for the significance of the differences were performed by ANOVA, followed by Tukey’s pairwise multiple comparison of means (GraphPad Prism Software). Data shown are averages ±SD, and changes in question were considered to be significant at *P* < 0.001.

### Supplementary Information

**Additional file 1 **: **Fig. S1** Overall structure of Marker Free binary expression vector of Reporter genes and PTG/Cas9 system. **Fig. S2** GUS Staining of transgenic hairy roots from *Actinidia chinensis* ‘Hongyang’. **Fig. S3** CaCl_2_ treatment increase the formation of calcium oxalate crystals in the root tip.

## Data Availability

All relevant data supporting the findings of this study are available within the paper. The vectors used in this study are available from the corresponding author, upon request.

## Abbreviations

CaOx: Calcium Oxalate
A.rhizogenes: *Agrobacterium rhizogenes*
A.tumefaciens: *Agrobacterium tumefaciens*
ArMT: *Agrobacterium rhizogenes*-mediated transformation
*CRISPR*: Clustered regularly interspaced short palindromic repeats
Cas9: CRISPR-associated protein 9
gRNA: Guide RNA
PTG: Polycistronic tRNA-gRNA
*GUS*: *β-glucuronidase*
*eGFP*: *Enhanced Green Fluorescent Protein*
*CBL3*: *Calcineurin B-like 3*
*CEN*: *CENTRORADIALIS*
*A.eriantha*: *Actinidia eriantha*
*A.chinensis*: *Actinidia chinensis*
