# Supplementary material for: Agrobacterium rhizogenes-mediated marker-free transformation and gene editing system revealed that AeCBL3 mediates the formation of calcium oxalate crystal in kiwifruit
Source: Mol Hortic. 2024 Jan 2;4:1. doi: 10.1186/s43897-023-00077-w (PMC10759683; doi:10.1186/s43897-023-00077-w)
Supplement: Supplementary file 1 — Additional file 1 : Fig. S1 Overall structure of Marker Free binary expression vector of Reporter genes and PTG/Cas9 system. Fig. S2 GUS Staining of transgenic hairy roots from Actinidia chinensis ‘Hongyang’. Fig. S3 CaCl2 treatment increase the formation of calcium oxalate crystals in the root tip. [file 43897_2023_77_MOESM1_ESM.pdf]

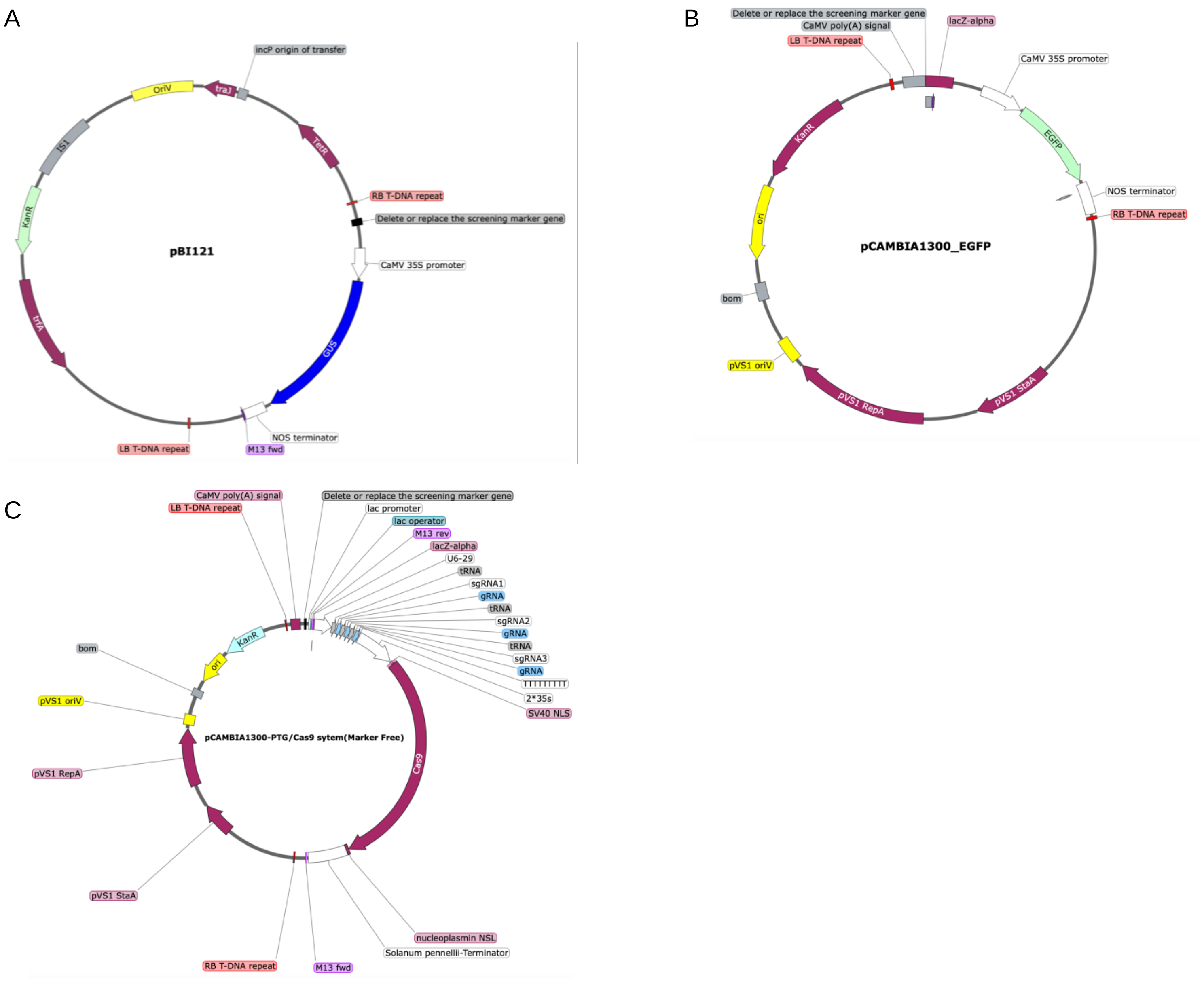

Figure S1 Overall structure of Marker Free binary expression vector of Reporter genes and PTG/Cas9 system. (A) pBI121-GUS. (B)pCAMBIA1300-EGFP. (C) Overall structure of the Cas9 binary expression vector pCAMBIA1300-PTG/Cas9.

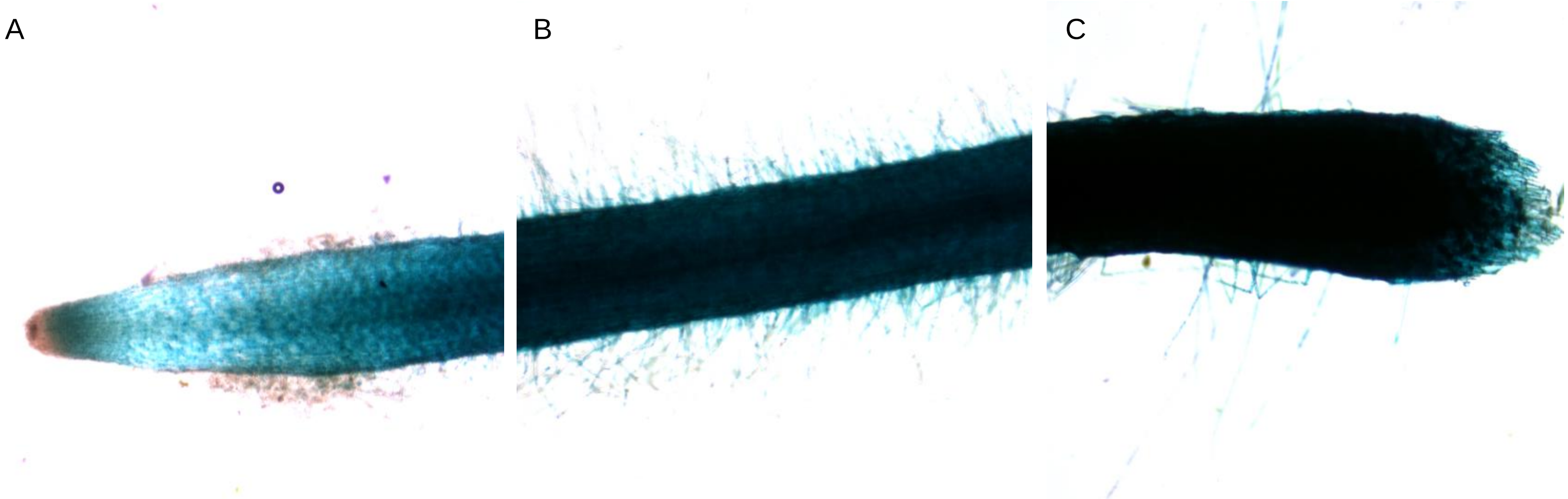

Figure S2 GUS Staining of transgenic hairy roots from *Actinidia chinensis* ‘Hongyang’. (A)(B)(C) represents different parts of the hairy root.

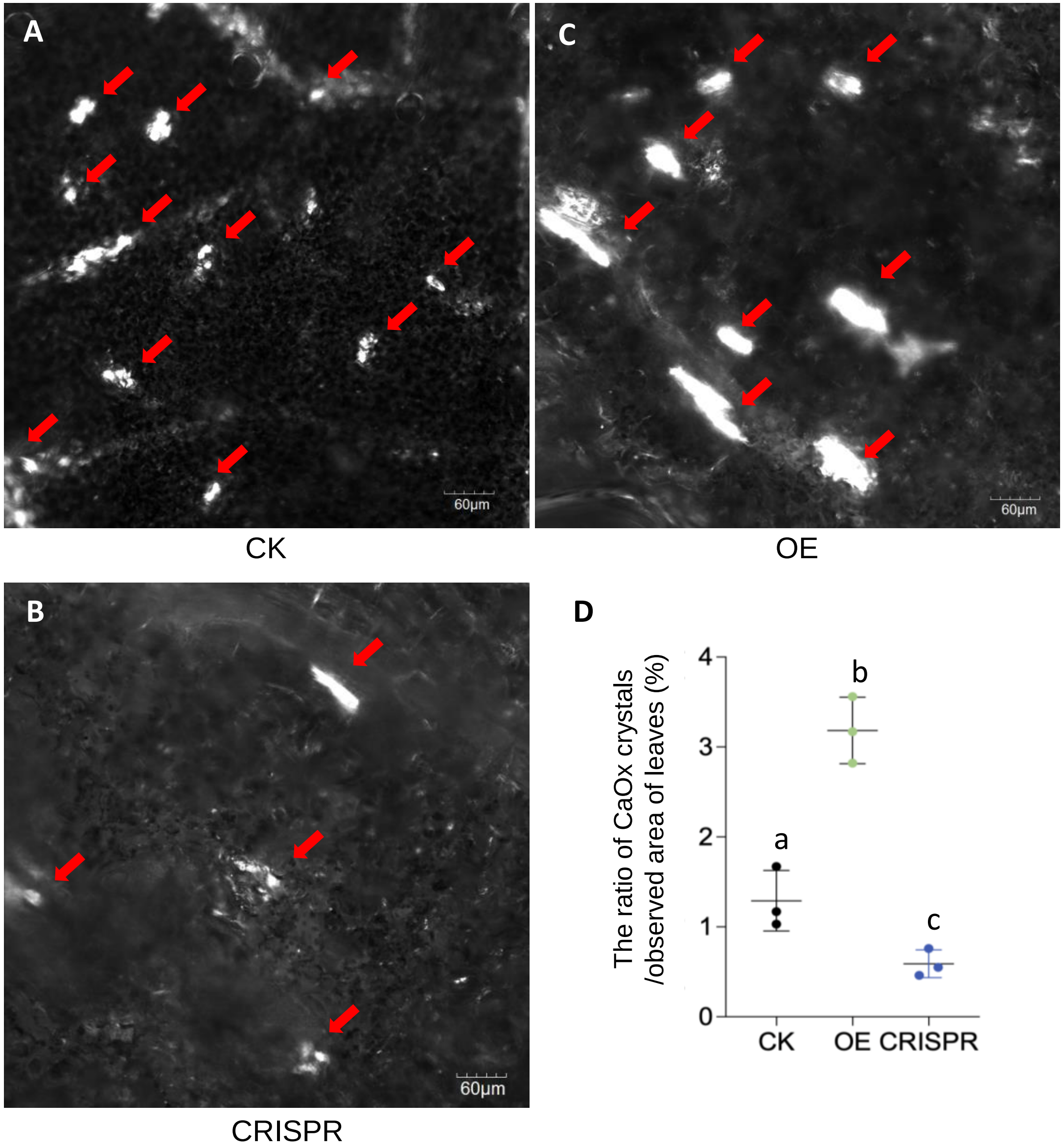

Figure S3 The observation of calcium oxalate (CaOx) crystals in the leaves.
(**A**-**C**) The observation of calcium oxalate crystals in the independent lines of Control, CK (**A**), CRISPR/Cas9 knockout lines, CRISPR (**B**), and Overexpression, OE (**C**) using polarized light on confocal microscope. The bright blocks indicated by red arrows are CaOx crystals. (**D**) The area of CaOx crystals were measured by ImageJ. The ratios of CaOx crystal area/ the observed area of leaves were represented by black dots, green dots and blue dots, respectively. Data shown are averages ±SD; n = 3. Significant differences compared with each other according to two-way ANOVA followed by Tukey's multiple comparison test are indicated with a, b and c (P < 0.001).
